# P-1550. Early Infant Gut Dysbiosis Associated with Extended-Spectrum Beta-Lactamase Producing Escherichia coli (ESBL-EC) Colonization in a Low-Resource Setting

**DOI:** 10.1093/ofid/ofaf695.1730

**Published:** 2026-01-11

**Authors:** Mehreen Arshad, Noelle Samia, Naveed Iqbal, Aneela Pasha, Umar Amjid, Romesa Ibrahim, Waqasuddin Khan, Imran Nisar, Fyezah Jehan

**Affiliations:** Lurie Children's/Northwestern, Chicago, Illinois; Northwestern University, Evanston, Illinois; Aga Khan University Hospital, Karachi, Sindh, Pakistan; Aga Khan University Hospital, Karachi, Sindh, Pakistan; Aga Khan University Hospital, Karachi, Sindh, Pakistan; Aga Khan University Hospital, Karachi, Sindh, Pakistan; Aga Khan University Hospital, Karachi, Sindh, Pakistan; Aga Khan University Hospital, Karachi, Sindh, Pakistan; Aga Khan University Hospital, Karachi, Sindh, Pakistan

## Abstract

**Background:**

*In vitro* and *in vivo* studies have shown that Extended-Spectrum Beta-Lactamase-producing *Escherichia coli (*ESBL-EC) can out-compete non-resistant commensals. The rapidly developing infant gut microbiome is susceptible to colonization with ESBL-EC, which may displace commensals leading to dysbiosis. Gut dysbiosis is linked to growth impairment in infancy, especially in low-resource settings. We aimed to investigate the interplay of ESBL-EC colonization, gut microbiome and infant growth among Pakistani mother-infant dyads.

**Figure 1:**
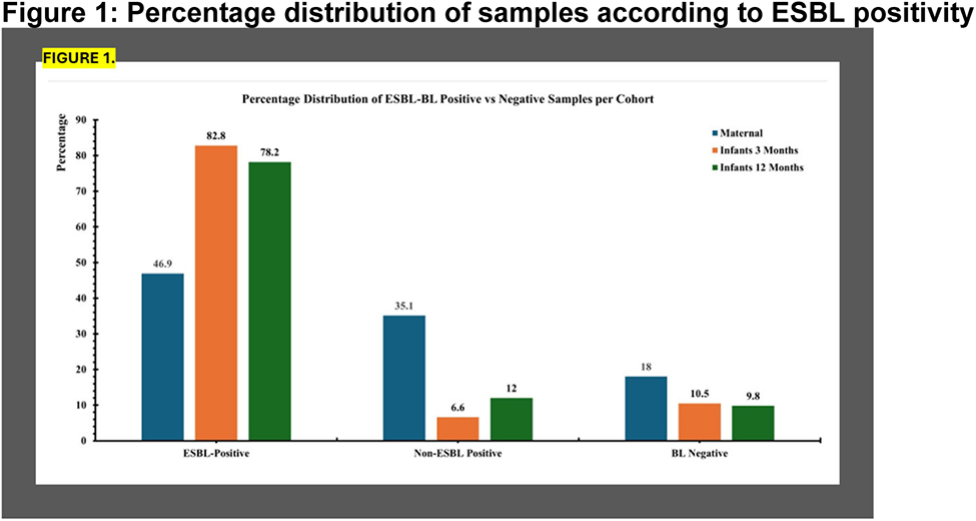
Percentage distribution of samples according to ESBL positivity The majority of infant samples were positive for ESBL genes (ESBL positive). Whereas most maternal samples either carried non-ESBL beta-lactamases (Non-ESBL positive) or carried no beta-lactamases (BL negative).

**Figure 2:**
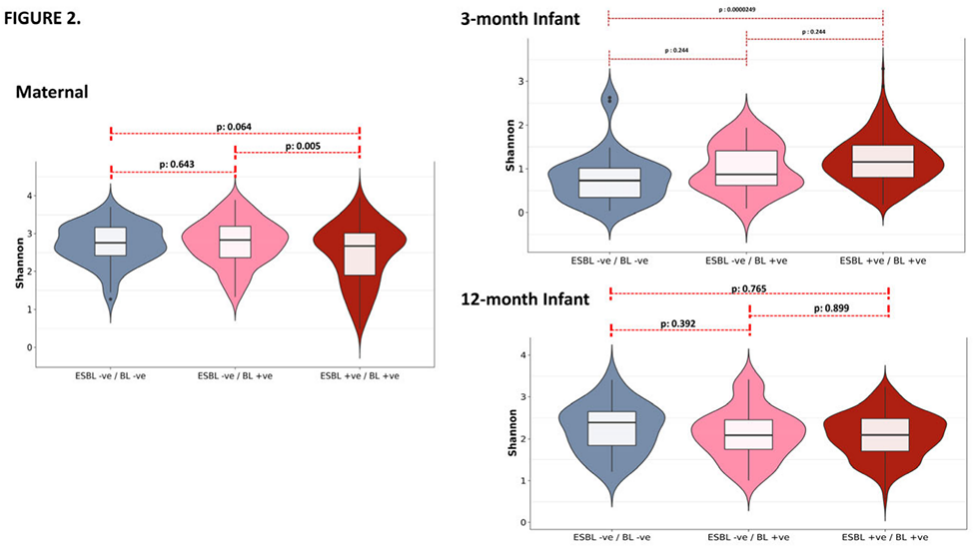
Shannon Diversity Index Shannon diversity index of microbial communities in relation to the presence or absence of ESBL and non-ESBL beta-lactamase genes among maternal and infants samples is shown.

**Methods:**

We utilized the Alliance for Maternal and Newborn Health Improvement (AMANHI) biorepository, which enrolled participants in a Pakistani community. Maternal and 3- and 12-month infant stool from 350 dyads underwent shotgun sequencing. Anthropometry was recorded, z-scores calculated, and odds ratios estimated using the elrm package in R.

**Figure 3:**
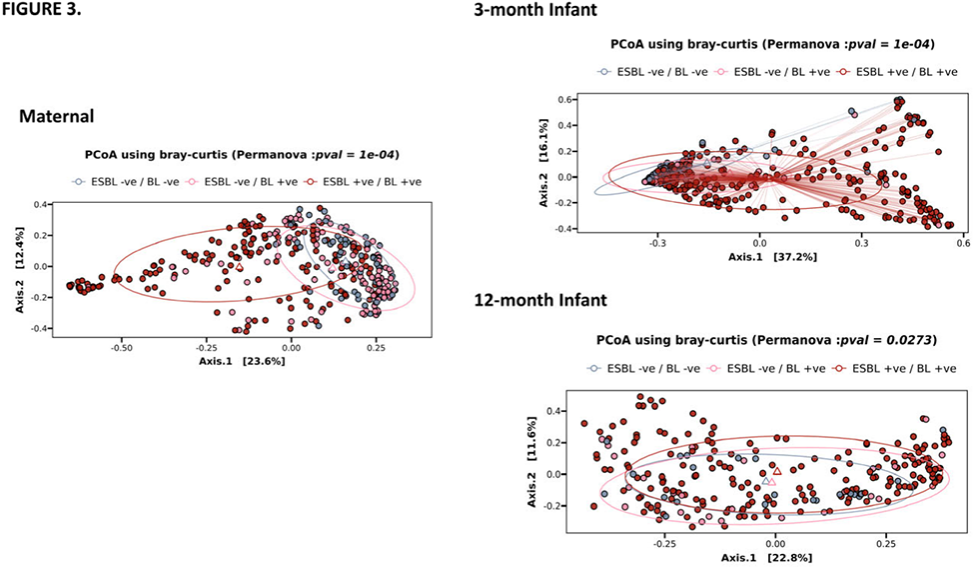
Principal Coordinates Analysis (PCoA) plot for β-diversity using the Bray-Curtis dissimilarity metric Maternal and 3-month infant ESBL positive samples showed a distinct pattern of clustering compared to the other non-ESBL BL positive samples and BL negative samples. At 12-months there was greater overlap between the three groups.

**Results:**

ESBL gene positivity (ESBL-pos) among maternal and 3- and 12-month infants was 46.9%, 82.8%, and 78.2%, respectively (Figure 1). Maternal ESBL-pos samples had significantly lower α-diversity compared to non-ESBL BL samples (p=0.005), while 3-month infant ESBL-pos samples had higher diversity than BL negative samples (p=< 0.001). In contrast, among 12-month infants, no significant difference was observed between any group (Figure 2). Principal Coordinates Analysis (PCoA) plots for β-diversity revealed distinct clustering of ESBL-pos samples among mothers and their 3-month-old infants compared to the non-ESBL BL and BL-neg groups (PERMANOVA p = 1e-04). By 12-months, we noted greater overlap these groups, though the clusters remained significantly different (PERMANOVA p = 0.0273, Figure 3). In both maternal and infant samples, ESBL-pos was associated with significant increase in *E. coli* abundance and decrease in commensals such as *Prevotella copri* and *Bifidobacterium longum*. After controlling for confounders, the odds of an infant born to ESBL-pos mothers being underweight at 3 months was 3.47 times that of an infant born to ESBL-neg mothers (95% Confidence Interval: (1.01, 19.3); p = 0.03).

**Conclusion:**

ESBL-positivity is associated with increased *E. coli* abundance, reduced commensals, altered gut microbial diversity, and underweight status in infants.

**Disclosures:**

All Authors: No reported disclosures

